# Health Economic Assessment: A Methodological Primer

**DOI:** 10.3390/ijerph6122950

**Published:** 2009-11-27

**Authors:** Steven Simoens

**Affiliations:** Research Centre for Pharmaceutical Care and Pharmaco-economics, Faculty of Pharmaceutical Sciences, Katholieke Universiteit Leuven, Onderwijs en Navorsing 2, P.O. Box 521, Herestraat 49, 3000 Leuven, Belgium; E-Mail: steven.simoens@pharm.kuleuven.be; Tel.: +32-16-323465; Fax: +32-16-323468

**Keywords:** health economics, health technology, cost study, economic evaluation, budget impact analysis

## Abstract

This review article aims to provide an introduction to the methodology of health economic assessment of a health technology. Attention is paid to defining the fundamental concepts and terms that are relevant to health economic assessments. The article describes the methodology underlying a cost study (identification, measurement and valuation of resource use, calculation of costs), an economic evaluation (type of economic evaluation, the cost-effectiveness plane, trial- and model-based economic evaluation, discounting, sensitivity analysis, incremental analysis), and a budget impact analysis. Key references are provided for those readers who wish a more advanced understanding of health economic assessments.

## 1. Introduction

Over the past decades, health technologies have made a major contribution to improving the health status of populations [1]. At the same time, during the 1995–2005 period the countries of the Organization for Economic Co-operation and Development (OECD) have witnessed an annual average health expenditure growth *per capita* of 4%. Growth in health expenditures outpaced the economic growth of 2.2% during the same period. Health expenditure growth can be attributed to a number of factors, including ageing populations, the increasing prevalence of chronic conditions, health care resource use price inflation, technological advances, and increased expenditures on drugs. With respect to the latter, annual average growth in pharmaceutical expenditure *per capita* of 4.6% during the 1995–2005 period exceeded the annual rise in health expenditures [2].

In response to this, governments seek instruments that can aid the implementation of safe and effective health technologies that support further health improvements, whilst containing health expenditures. Health economic assessment provides a tool to maximize population health subject to scarce resources [3] and to consider the extent to which a technology will be used. Furthermore, evidence derived from health economic assessments is used to inform decisions about the registration, pricing/reimbursement of health technologies in an increasing number of countries [4]. The requirement for health economic assessment fits within an overall trend towards evidence-based decision making in health care [5].

The aim of this article is to provide an introduction to the science underlying the health economic assessment of a health technology. This article serves as a resource for readers who want a succinct overview of the methodology and use of health economic assessment. Particular attention is paid to defining the fundamental concepts and terms that are relevant to health economic assessment. Key references are added for those readers who wish a more advanced understanding of these topics.

## 2. Health Economics (Assessment)

Health economics as a scientific discipline that applies economic principles to health and health care [6]. Health economic aspects include, amongst other things, health policy and regulation, the organization and financing of health care, international comparisons of health care systems, the supply of and demand for health care, inequalities in health, the supply of and demand for health insurance. One specific aspect of health economics involves the assessment of a health technology. Such an assessment may consist of a cost study, a budget impact analysis and/or an economic evaluation. This article focuses on these three health economic assessment techniques.

## 3. Cost Study

A cost study can serve multiple purposes. Cost estimates can underline the importance of a disease to society when considered alongside its impact on morbidity and mortality and when compared with the economic burden of other diseases. Furthermore, cost studies may allow the identification of the drivers of diagnosis and treatment costs. Finally, cost data can be fed into economic evaluations, so that decision makers can ascertain the efficiency of various approaches to diagnosing and treating a disease by examining their consequences in relation to their costs.

Information about costs can be derived from a cost-of-illness analysis or from a cost analysis. A cost-of-illness analysis quantifies the economic burden of a disease to society by measuring the costs of diagnosing and treating a disease, as well as the costs arising as a result of the disease (for instance, productivity losses due to time taken off work). A cost analysis compares the costs of two or more approaches to diagnosis and treatment of a disease (for instance, medical *versus* surgical therapy).

The following categories of costs can be distinguished [7]: direct costs refer to the costs of health care services such as costs of drugs, physician visits and hospitalisation. If health care services keep a patient alive, the patient is likely to fall ill in the future and require additional health care services. Health care costs in the added years of life as a result of keeping patients alive may also be counted as direct costs. Direct costs sometimes include patients’ out-of-pocket expenses such as the costs of transportation to the hospital and the costs of child care while the patient is receiving treatment. Indirect costs reflect the costs of productivity loss as a result of the disease. These costs not only consist of the productivity loss of the patient, but also of the productivity loss of family or friends who take time off work to care for the patient. The productivity loss may take the form of time lost from work (‘absenteeism’) or reduced productivity at work (‘presenteeism’).

Costs originate in the health care sector and in other sectors. For instance, treatment of opiate-dependent drug users involves the health sector (through the provision of maintenance or detoxification programmes), but also relies on the input from social care agencies. Furthermore, some studies have demonstrated that treatment costs are offset by savings arising from the prevention of future health care use and the reduction in criminal justice expenditure [8]. Costs are incurred by the health care payer (*i.e.*, insurance funds or national health service), the patient/family (e.g., drug co-payment, costs of home adaptation) and by the society at large (e.g., costs of productivity loss).

Once the relevant resource use has been identified, measured and valued, costs can be calculated [9,10]. These four steps are now described in detail in the following sections.

### 3.1. Identification of Resource Use

The perspective of the study determines which items of resource use need to be taken into account. A cost study can take a societal perspective by considering all (in)direct (non-)health care resource uses. Alternatively, the more narrow perspective of a Ministry of Health, health care payer, hospital or patient can be adopted. In these instances, the cost study considers those items of resource use that are relevant from the perspective of the study. For instance, productivity loss as a result of illness is included in a cost study from the societal perspective. However, productivity loss is not relevant to the Ministry of Health and is, thus, excluded from a study with such a perspective.

The time horizon of a cost study needs to cover all relevant resource use. This applies to cost analyses, particularly those of immunization or vaccination programmes. Such programmes are associated with the use of drugs in the short-term, but may lead to savings from reduced health care resource use and from less productivity loss in the future. The time horizon needs to be sufficiently long to be able to investigate whether present drug costs are offset by future cost savings. The time horizon is also relevant to cost-of-illness analyses. A cost-of-illness analysis may take the form of a prevalence-based study, which measures costs attributable to a group of patients suffering from a disease during a given time interval. For instance, a literature review indicated that cost-of-illness analyses of endometriosis measured costs during a time period varying from six months to five years. This period was too short to account for the chronic nature of endometriosis which may afflict women during their reproductive years [11]. Therefore, cost-of-illness analyses need to take the form of an incidence-based study, quantifying costs of a disease from onset to end.

### 3.2. Measurement of Resource Use

Two approaches can be adopted to measure the volume of resource use. On the one hand, a micro-costing or bottom-up approach identifies and measures each relevant item of resource use. This approach generates estimates of resource use with a high level of precision. However, this approach is time-intensive, expensive, and may yield estimates that are context-specific. On the other hand, a gross-costing or top-down approach measures resource use at the aggregate level (e.g., at the level of diagnosis-related groups) without specifying individual items. Such estimates benefit from increased generalisability and improve comparability of cost studies, but are less precise.

In terms of data sources, resource use can be measured in a sample of patients (primary data collection). A cost study can follow up patients suffering from a specific disease. Such case series that focus on identified patients only may be misleading in the case of diseases where diagnosis is complex and attribution of resource use to the disease is difficult. Studies comparing patients with/without a disease are better suited in that they allow identification of additional resource use related to the disease. Resource use can also be derived from existing sources such as patient medical records, a health care payer claims database, the published literature, other routine data sources or large scale secondary datasets (secondary data collection). Patient medical records provide detailed information about health care resource use. On the one hand, such data tend to pertain to a specific institution(s), thus limiting the generalisability of cost estimates. On the other hand, it may be possible to extract medical record data from a representative patient sample or from a sample of patients from multiple institutions. An analysis of claims data benefits from comprehensiveness of information on health care resource use, but may suffer from missing data and incorrect diagnostic coding of claims. Resource use data can be gathered from the literature, although differences in the design of primary studies may restrict comparability of estimates. The key issue is that secondary data sources may not fit the question that a health economic assessment seeks to address.

### 3.3. Valuation of Resource Use

The principle of valuing resource use is based on the notion of ‘opportunity cost’. The opportunity cost represents the cost of using resources for some purpose, measured as their value in their next best alternative use. In the context of health economic assessment, the valuation of resource use puts a monetary value on the resources depleted by the disease and its treatment. Economic theory demonstrates that market prices in a free and perfectly competitive market represent opportunity costs. Thus, in order to value resource use, the volume of resource use needs to be multiplied by market prices. However, market prices do not always exist. For instance, drug prices may be negotiated between the government and the pharmaceutical company. Therefore, researchers use official list prices to calculate charges. Caution needs to be exercised when calculating charges as these do not necessarily reflect the worth of resource use. For instance, charges of surgical treatment in hospital may not accurately measure actual expenditure on administration, billing, capital depreciation, maintenance, laundry and other hospital services related to the surgical procedure.

Alternatively, shadow prices can be used to value resource use in the absence of market prices. This can be illustrated with the valuation of productivity loss. If the patient is an employee, his/her wage can be used to value lost productivity. If the patient is a housewife, this approach cannot be used as a housewife does not receive a wage. Instead, researchers need to draw on a shadow price, *i.e.*, the market price of a similar activity. In this example, the market wage of a professional housekeeper could serve as the shadow price and could be used to value the productivity loss of the housewife.

### 3.4. Calculation of Costs

When calculating the costs of a health care programme, the question arises of whether to compute marginal or average costs. Dividing total costs by the number of units generates average costs. Average costs include fixed costs (e.g., costs of hospital infrastructure) as well as variable costs. Marginal costs represent the costs of producing one additional unit and, therefore, include variable costs only. As our interest is in the additional costs incurred by the health care programme, a cost study needs to calculate marginal costs. However, the distinction between average and marginal costs is not always clear. If the national implementation of a health care programme involves building a new hospital, the use of average costs is recommended as they measure the fixed costs of the hospital infrastructure. Alternatively, these hospital costs can be viewed as marginal costs as they represent the additional costs imposed by the programme.

## 4. Economic Evaluation

**Figure 1.**
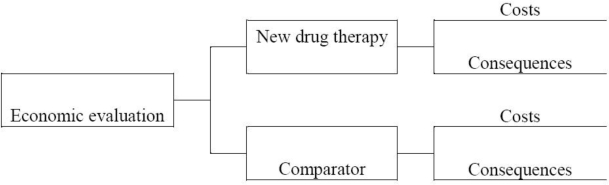
Components of an economic evaluation.

An economic evaluation is in essence a comparative analysis of at least two health technologies in terms of both their costs and consequences [7]. Figure 1 portrays the components of an economic evaluation of a new drug therapy vis-à-vis a comparator. The comparator is generally chosen to reflect common clinical practice in the setting where the economic evaluation is undertaken. In our example of a new drug therapy, the comparator can be an older drug or another health technology. If the new drug represents the first technology that is available to treat a specific disease, the relevant comparator may be no active therapy.

An economic evaluation enables us to answer efficiency questions by relating the costs to the consequences of alternative health technologies. An incremental analysis is carried out to express the results of an economic evaluation. This means that, for meaningful comparison, an economic evaluation expresses the additional costs incurred by one health technology vis-à-vis the comparator in relation to the additional consequences of that technology vis-à-vis the comparator.

### 4.1. Types of Economic Evaluation

An economic evaluation can take a number of forms. A cost-minimisation analysis is appropriate when the alternative health technologies produce equivalent consequences. In this case, a cost-minimisation analysis identifies the least costly health technology. For instance, an economic evaluation examined the efficiency of two antibiotics (teicoplanin and vancomycin) used in the treatment of intensive care unit patients with catheter-related infections [12]. Comparative trials of teicoplanin and vancomycin reported no significant differences in their consequences (*i.e.*, effectiveness and safety). Therefore, a cost-minimisation analysis compared costs of drug acquisition, materials required for preparation and administration of antibiotics, laboratory tests and nursing time. Treatment with teicoplanin turned out to be more expensive than vancomycin. This was because lower costs of laboratory tests with teicoplanin only partially offset higher drug acquisition costs.

A cost-effectiveness analysis denotes an economic evaluation that measures costs in a monetary unit and quantifies a single consequence in a physical or natural unit (e.g., the number of successfully treated patients, the number of life years gained, the number of symptom days averted). Final consequence measures (e.g., life years gained) are preferred to intermediate measures (e.g., cholesterol level) as our interest is in the ultimate impact of a technology on health. Also, as final consequence measures are relevant to multiple health technologies and diseases, their use facilitates comparison of the efficiency of various technologies. The results of a cost-effectiveness analysis are expressed by means of an incremental cost-effectiveness ratio:

$$\text{Incremental}\;\text{cost}-\text{effectiveness}\;\text{ration}\;=\;({\text{C}}_{1}-{\text{C}}_{0})/({\text{E}}_{1}-{\text{E}}_{0})$$

where C_1_ is the cost of the health technology; C_0_ is the cost of the comparator technology; E_1_ and E_0_ are the consequences of the technology and the comparator, respectively.

A cost-effectiveness analysis is only possible if the health technologies affect the same consequence. However, technologies may affect multiple consequences. In this case, a cost-consequence analysis can be carried out, which presents costs and multiple consequences. One use of a cost-consequence analysis is to highlight the choices and trade-offs that exist between different consequences. The drawback is that a cost-consequence analysis does not provide an assessment of the overall efficiency of a health technology.

The previous types of economic evaluation pose a number of challenges. In particular, health technologies may impact multiple consequences, consequences may differ between health technologies, and patients may attach more importance to some consequences than others. In response to this, the two following types of economic evaluation have been developed that combine and value the various consequences in a single generic measure of health improvement.

An economic evaluation that measures costs and consequences by means of specific health-related quality of life measures, such as quality-adjusted life years, is referred to as a cost-utility analysis. The quality-adjusted life year takes into account the quantity and quality of life. The quality of life associated with a health state is measured through the use of health utilities. A utility reflects the preference of an individual for the health state. Utilities are elicited on a scale of 0 (reflecting death) to 1 (reflecting perfect health) using techniques such as the visual analogue scale, the standard gamble or the time trade-off [13]. Quality of life data are then combined with estimates of the time period for which the health benefits last to generate quality-adjusted life years. The results of a cost-utility analysis using quality-adjusted life years are expressed by means of an incremental cost-utility ratio:

$$\text{Incremental}\;\text{cost}-\text{utility}\;\text{ration}\;=\;({\text{C}}_{1}-{\text{C}}_{0})/({\text{Q}}_{1}-{\text{Q}}_{0})$$

where C_1_ is the cost of the health technology; C_0_ is the cost of the comparator technology; Q_1_ and Q_0_ are the number of quality-adjusted life years associated with the technology and the comparator, respectively.

A cost-benefit analysis values consequences in monetary terms (the ‘benefits’) in addition to costs. A monetary value can be assigned to consequences by means of the human capital approach, the revealed preference approach or the willingness-to-pay technique [14]. However, assigning monetary values to consequences is controversial and further work on methods to value consequences needs to be carried out. As both costs and consequences are expressed in monetary terms, costs can be directly compared with benefits and the net worth (benefits minus costs) of a health technology can be estimated. The results of a cost-benefit analysis may be stated in the form of the net benefit (or net loss) of one health technology over another or in the form of an incremental cost-benefit ratio:

$$\begin{array}{c} \text{Net}\;\text{benefit}\;=\;({\text{B}}_{1}-\;{\text{B}}_{0})-({\text{C}}_{1}-{\text{C}}_{0})\\ \text{Incremental}\;\text{cost}-\text{benefit}\;\text{ratio}\;=\;({\text{C}}_{1}-{\text{C}}_{0})/({\text{B}}_{1}-{\text{B}}_{0}) \end{array}$$

where C_1_ is the cost of the health technology; C_0_ is the cost of the comparator technology; B_1_ and B_0_ are the benefits of the technology and the comparator, respectively.

### 4.2. The Cost-Effectiveness Plane

**Figure 2.**
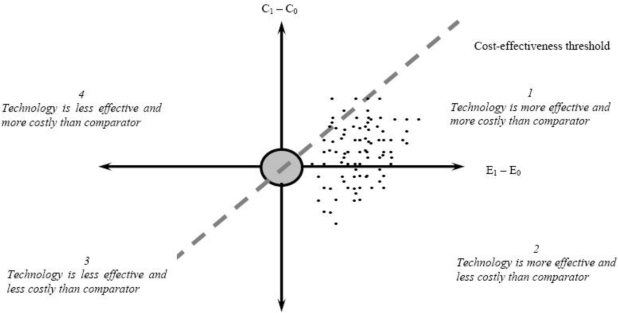
The cost-effectiveness plane.

The question whether to conduct an economic evaluation can be answered by looking at the so-called cost-effectiveness plane (see Figure 2) [15]. On the horizontal axis, the difference in effectiveness (e.g., life years) between the health technology and the comparator is portrayed. The vertical axis represents the cost difference between the technology and the comparator. The technology may have higher or lower costs, and higher or lower effectiveness than the comparator, so that its point may fall into one of the four quadrants.

If the point falls into quadrant 2, the technology is more effective and less costly than the comparator. In other words, the technology dominates the comparator. This indicates that the technology needs to be adopted and that there is no need to conduct an economic evaluation. Conversely, if the point falls into quadrant 4, the comparator dominates the technology and the comparator should be adopted. In quadrants 1 and 3, one option is more effective, but also more costly than the other option. In these cases, an economic evaluation needs to be carried out. Authorities in some countries have specified cost-effectiveness thresholds, which serve to determine whether a technology is efficient or not (cfr. infra). Such a threshold represents the maximum cost per life-year gained that authorities are willing to pay for a health technology. The gradient of the dashed line represents one cost-effectiveness threshold. The technology is efficient if its point falls to the south-east of this dashed line.

What is the empirical evidence of the value of health technologies? A recent study reviewed 599 articles providing data on 1,500 cost-effectiveness ratios of health technologies [16]. Technologies included preventive measures (interventions designed to avert disease or injury) and curative measures (interventions designed to reverse or retard progression of an existing condition and interventions designed to ameliorate the effects of a disease). The distribution of preventive measures spanned the full range of cost-effectiveness results: preventive measures included dominant measures, measures with a positive incremental cost-effectiveness ratio and dominated measures. In fact, preventive measures and curative measures exhibited a similar distribution of cost-effectiveness ratios.

### 4.3. Trial- and Model-Based Economic Evaluation

There are two ways to carry out an economic evaluation: a trial-based economic evaluation or a model-based economic evaluation [17].

An economic evaluation can be carried out alongside a clinical trial. Such evaluations are called trial-based economic evaluations or piggy-back studies. In the case of a piggy-back study of a new drug, the economic evaluation can be carried out alongside a Phase III clinical trial, which examines the efficacy and adverse reactions during the drug development process. Such economic evaluations provide timely information with high internal validity that can be used by manufacturers, policy makers, healthcare professionals and patients to assess the value of a new drug. The economic evaluation can also be conducted alongside a Phase IV clinical trial, which examines long-term effectiveness following regulatory approval of the drug. Such economic evaluations explore the efficiency of a new drug under conditions of day-to-day practice and benefit from greater external validity.

There is a wide diversity in the design, conduct and analysis of trial-based economic evaluations. However, a number of good research practices have emerged [18]. A gold standard trial-based economic evaluation should have adequate power with a view to testing hypotheses about expected differences in costs and consequences. An adequate time horizon needs to capture the long-term impact of the health technology. The choice of consequence measures in the trial must be suited for use in the economic evaluation. For instance, quality-of-life values derived from the trial can be used to calculate quality-adjusted life years in a cost-utility analysis. The identification, collection and management of economic data should be fully integrated into the clinical trial. Data analysis should follow an intention-to-treat approach, assess uncertainty, take account of time preference for costs and consequences, and account for missing or censored data. Appropriate summary measures need to be used to calculate the relative value of the technology vis-à-vis the comparator.

Nevertheless, there are some drawbacks to using piggy-back studies for the purpose of economic evaluation. These include:

- Inadequate sample size;
- Restrictive patient selection (patient characteristics, co-morbidities, disease severity);
- Inappropriate comparator;
- Short time horizon;
- Occurrence of protocol-driven resource use (which leads to over-estimation of costs);
- Artificially enhanced compliance;
- Inappropriate consequence measures;

It needs to be emphasised that trials are conducted for a wide variety of purposes (e.g., dose response, safety, efficacy, effectiveness) and as such may or may not be suitable for consideration of cost-effectiveness as an economic evaluation considers whether an intervention is worth adopting into practice. As a consequence some or all of the drawbacks may exist. They are less likely to exist in a study that is explicitly designed to address cost-effectiveness as a primary outcome.

Even if a trial-based economic evaluation exists and is suitable, some form of modeling is likely to be needed. For instance, to examine the full impact of a health technology, statistical modeling beyond the time horizon of the trial may be required for the purpose of extrapolation. Also, decision-analytic modeling may be used to address some shortcomings of trial-based economic evaluations by allowing us to compare all relevant options; to incorporate all appropriate evidence; to translate intermediate endpoints into final consequences; to extrapolate over the appropriate time horizon of the evaluation; and to generalize to other settings or populations [19]. Decision-analytic modeling is the main modeling approach used in health economic assessment and is, in essence, a quantitative approach to decision making under conditions of uncertainty. A model can be defined as an analytic methodology that accounts for events over time with a view to estimating the impact of a health technology on costs and consequences [20]. Decision-analytic modeling can take the form of a decision tree or a Markov model. A decision tree is a graphic representation of the various diagnosis and treatment pathways of a specific disease in combination with the probabilities, costs and consequences associated with each pathway. A Markov model structures a disease and its treatment process by means of mutually exclusive and exhaustive health states, with patients moving from one health state to another based on transition probabilities. The time spent in a health state generates costs and consequences. Estimates of probabilities, costs and consequences used in decision trees and Markov models are usually derived from the literature or from expert opinion.

With respect to modeling, concerns have been raised about the inappropriate use of clinical data, about biases in observational data, about the difficulties of extrapolation, and about the transparency or validity of models [19]. Therefore, it is important to adhere to principles of good practice for modeling in economic evaluation by ensuring that the model represents the key features of the decision to be made; by presenting the results in a transparent way; by respecting the quality of the data used in the model; and by exploring uncertainty. Attention also needs to be paid to validating the model by comparing the results with those of similar studies. Finally, the reader should note that a model is only as good (or bad) as the quality of its data and its specification. Thus, model-based economic evaluations need to clearly state the caveat that the results are conditional on the data and assumptions incorporated in the model.

### 4.4. Discounting

The costs and consequences of a health technology generally do not take place in the same year, but may be spread out over multiple years. For instance, current costs of a vaccination programme need to be compared by future benefits of prevented disease and reduced healthcare costs. An economic evaluation needs to take account of the timing of costs and consequences because individuals have a positive rate of time preference. This means that individuals attach greater importance to current than to future costs and consequences. This positive rate of time preference mainly derives from three reasons: (a) individuals consider the short run only; (b) individuals are uncertain about the future; and (c) individuals can invest a Euro now and expect to receive more than a Euro in the future. Time preference is taken into account in an economic evaluation by the process of discounting. Discounting calculates the present value of costs and consequences occurring in the future [21]. By calculating present values, alternative health technologies with differential timing of costs and consequences can be compared from the same baseline.

### 4.5. Sensitivity Analysis

Any variable used in an economic evaluation is subject to some uncertainty [22]. This uncertainty can originate from methodological disagreements, researchers’ assumptions in the absence of data, imprecise data, need to extrapolate results over time, and the need to generalize results to other settings or other countries. A sensitivity analysis determines the direction and the extent to which the results of the economic evaluation vary when estimates of input variables change.

There are two approaches to carrying out a sensitivity analysis in a model-based economic evaluation: deterministic and probabilistic sensitivity analysis.

A deterministic sensitivity analysis explores the impact on results of changes in one input variable (one-way analysis) or of simultaneous changes in multiple variables (multi-way analysis). One application of a multi-way analysis is a scenario analysis. Such an analysis typically includes a best case scenario, where all input variables are changed in the most optimistic way, and a worst case scenario, where input variables take on the most pessimistic values. A scenario analysis provides insight into the efficiency of the health technology in the best case and in the worst case. Such an analysis may also serve to test the impact of various scenarios on cost-effectiveness results. Finally, a threshold analysis identifies the combination of variable estimates that ensures that the incremental cost-effectiveness or cost-utility ratio of the technology does not exceed the threshold adopted by authorities.

A probabilistic sensitivity analysis is based on a Monte Carlo simulation. The principle is to run the analysis a large number of times (e.g., 10,000 times) with different sets of variable estimates drawn from distributions. This requires that a probability distribution is assigned to each input variable. For each iteration, the simulation draws input parameters at random from their statistical distributions and calculates cost and effectiveness pairs. At the end of the 10,000 iterations, the joint statistical distribution for costs and effectiveness is represented as a cloud of points on the cost-effectiveness plane (see Figure 2).

**Figure 3.**
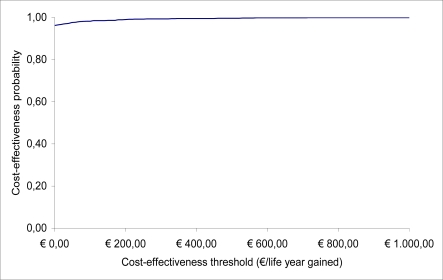
Cost-effectiveness acceptability curve.

The gradient of the dashed line in Figure 2 indicates one cost-effectiveness threshold. Typically, this line cuts through the cloud of cost and effectiveness pairs generated by the probabilistic sensitivity analysis. Simulations falling to the south-east of the line support the cost-effectiveness of the health technology. The probability that the technology is cost-effective is estimated as the proportion of points to the south-east of this line. As the cost-effectiveness threshold increases, the dashed line rotates anti-clockwise around the origin, increasing the proportion of points to the right of the line. This allows us to draw cost-effectiveness acceptability curves representing the probability that the health technology is efficient for a range of cost-effectiveness thresholds (see Figure 3) [23].

A trial-based economic evaluation of patient-level data is inherently stochastic [22]. To account for uncertainty, confidence intervals for cost-effectiveness ratios can be estimated when the health technology is more effective and less costly than the comparator (quadrant 1 of the cost-effectiveness plane, see Figure 2). Multiple methods exist to estimate confidence intervals including the confidence box, Taylor series expansion, confidence ellipse, angular transformation, Fieller’s method and bootstrapping. When uncertainty covers more than one quadrant of the cost-effectiveness plane, a more general approach involves the calculation of net benefits, *i.e.*, the additional costs of a technology vis-à-vis the comparator divided by a specific cost-effectiveness threshold value. This can be followed by the presentation of results in a cost-effectiveness acceptability curve.

### 4.6. Use of Economic Evaluation by Decision Makers

**Figure 4.**
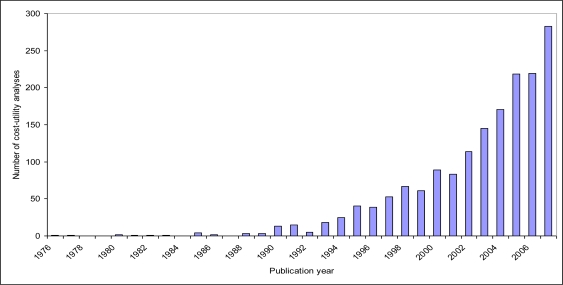
Trend in cost-utility analyses of health technologies, 1976–2007.

The use of economic evaluation is corroborated, for instance, by the dramatic increase in the number of published economic evaluations of health technologies in the last decades. For instance, the Tufts Cost-Effectiveness Analysis Registry, which includes more than 1,700 cost-utility analyses of health technologies published from 1976 to 2007, shows that the number of cost-utility analyses has risen exponentially over time (see Figure 4). Also, specific databases have been developed that contain information about economic evaluations of health technologies (e.g., the Health Economic Evaluations Database at McMaster University, Hamilton, ON, Canada) [24] and about the methodological quality of such economic evaluations (e.g., the National Health Service Economic Evaluation Database at the University of York, UK) [25].

As decision makers appreciate the need to evaluate projects in terms of costs and benefits, economic evaluation offers a framework that presents information about health technologies in a format that is familiar and useful to them. Economic evaluation may serve as an instrument to demonstrate the value of a health technology with a view to informing pricing/reimbursement decisions [26]. For this purpose, the results of a cost-effectiveness analysis or cost-utility analysis of a health technology may be compared with the cost-effectiveness threshold or cost-utility threshold, respectively, adopted by authorities. Health technologies with a cost-effectiveness/cost-utility ratio below the threshold are rewarded by means of a more favourable price/reimbursement.

Cost-effectiveness and cost-utility thresholds have either been explicitly specified by authorities or can be implicitly determined from examining past pricing/reimbursement decisions. Table 1 provides an overview of threshold values used to inform pricing/reimbursement decisions in Australia [27], Canada [28], England and Wales [29,30], The Netherlands [31], New Zealand [32], and the United States [33]. This Table shows that threshold values vary substantially between countries.

For instance, the National Institute for Health and Clinical Excellence (NICE) in England and Wales uses a threshold value of £20,000 per quality-adjusted life year, although health technologies with a cost-utility ratio above this threshold can be recommended for use in the National Health Service if there is a strong case to do so. A review of NICE guidance issued between 1999 and 2005 concluded that health technologies having a ratio exceeding £30,000 per QALY were unlikely to be recommended [29]. Judgements about what is regarded as an (un)acceptable cost-utility ratio are made by NICE's advisory committees, which consist of clinicians and health managers working in the National Health Service, statisticians, health economists, and patients [30]. However, there is a debate about whether the use of thresholds is informative, and alternative approaches to assess the value of a health technology have been proposed, such as the replacement approach, program budgeting and marginal analysis, generalized optimization framework, and multi-criteria decision analysis [34].

It should be noted that certain aspects of the decision making process restrict the use of economic evaluations of health technologies. A first aspect relates to institutional features of the health care system. For instance, in most European countries, health expenditures are divided across several budgets, with a tendency for decision makers to adopt a silo mentality. This means that decision makers consider each budget separately, but do not take account of the full impact of a technology across budgets. This silo mentality poses challenges for economic evaluation because health technologies are likely to have an impact on multiple budgets. For instance, although the introduction of a new drug may add to the pharmaceutical budget, this may be accompanied by reduced expenditure on other health services utilization. Therefore, there is a need to overcome this silo mentality in order to enhance the value of economic evaluation.

Economic evaluations need to report findings that have practical relevance to decision makers. For instance, savings arising from fewer hospitalizations are accounted for as a financial benefit in an economic evaluation. However, the benefit does not necessarily materialize in a real setting as vacated beds may be used in the treatment of other patients. If this is the case, researchers are essentially taking into account freed resources, whereas decision makers have an interest in actual financial savings. It is therefore important for researchers to understand the perspective of decision makers, to ascertain for what purpose decision makers wish to use the information derived from the economic evaluation, and to present the results accordingly.

Although decision makers embrace the principle of weighing costs and benefits in making decisions, their actual knowledge of economic evaluation techniques is generally limited and they tend to have doubts about the methodological quality of studies [35]. To overcome this barrier, there is a need for better education and training of decision makers in economic evaluation techniques. Moreover, a higher degree of standardization and consensus surrounding methodological principles of economic evaluation is required.

## 5. Budget Impact Analysis

In addition to information about the efficiency of a new health technology, regulatory agencies in an increasing number of countries now require data about the budgetary impact of the technology on national, regional or local budgets [36]. Whereas an economic evaluation allows decision makers to assess the efficiency of a health technology, a budget impact analysis examines the financial impact of the adoption and diffusion of the technology within a particular setting. Thus, a budget impact analysis considers the affordability of a technology. Specifically, a budget impact analysis explores how a change in the current mix of treatment strategies by the introduction of a new technology will impact spending on a disease.

Budget impact analysis in combination with cost study and economic evaluation play a crucial part in the comprehensive assessment of a health technology and may inform reimbursement decisions [26]. Reimbursement may be withheld from a cost-effective health technology if it has a high budgetary impact. Conversely, a cost-ineffective technology may receive reimbursement if its budgetary impact is limited. This is because the opportunity cost of adopting such a technology is low (little other activity would need to be displaced) and the adoption may meet other important objectives of a decision-maker such as equity. The reimbursement of orphan drugs, for instance, shows that decision makers may attach more importance to budget impact and equity considerations than to efficiency [37].

The methodology of budget impact analysis is still developing, although principles of good practice for budget impact analysis have recently been proposed [36]. A budget impact analysis starts with providing all relevant epidemiological, clinical and economic information of the disease. Then, the current mix of treatment strategies is described. This may cover no active therapy as well as therapies that may or may not be replaced by the new health technology. The introduction of the technology may lead to technology substitution and market expansion. Therefore, a budget impact analysis considers all patients who might be treated with the new technology, including previously untreated patients who may now seek treatment. Finally, the analysis considers the budgetary impact of various scenarios of how the current mix of treatment strategies changes when the new technology becomes available.

## 6. Conclusions

Health economic assessment in the form of a cost study, economic evaluation and budget impact analysis provides a tool to evaluate health technologies. Indeed, these instruments present information about the costs, efficiency and affordability of a technology to decision makers with a view to optimising health policy. In order to fully exploit the value of health economic assessment, researchers need to take care to conduct such exercises according to methodologically sound principles. Additionally, researchers need to take into account the decision making context by identifying the various goals that decision makers pursue and by discuss how decision makers can use the findings of health economic evaluation to attain these objectives.

## Figures and Tables

**Table 1.** Cost-effectiveness/cost-utility threshold values.

*Country*	*Threshold value in local currency*	*Threshold value in Euro*
Australia	AUS$42,000–76,000 per life year	24,700–44,700 € per life year
Canada	CAN$20,000–100,000 per QALY	12,700–63,300 € per QALY
England and Wales	£20,000–30,000 per QALY	22,800–34,100 € per QALY
Netherlands	20,000–80,000 € per QALY	20,000–80,000 € per QALY
New Zealand	NZ3,000–15,000 per QALY	1,400–7,200 € per QALY
United States	US$50,000 per QALY	34,400 € per QALY

Notes:

- QALY = quality-adjusted life year.

- Local threshold values were converted into Euro using market exchange rates on 14th September 2009
